# Informed consent and risk communication challenges in antimicrobial clinical trials: a scoping review

**DOI:** 10.1136/bmjopen-2023-082096

**Published:** 2024-11-24

**Authors:** Yiyun Shou, Joey Elizabeth Yeo, Alexander Shao-Rong Pang, David L. Paterson, Yin Mo

**Affiliations:** 1 Saw Swee Hock School of Public Health, National University of Singapore and National University Health System, Singapore; 2 Lloyd's Register Foundation Institute for the Public Understanding of Risk, National University of Singapore, Singapore; 3 School of Medicine and Psychology, Australian National University, Canberra, ACT, Australia; 4 Yong Loo Lin School of Medicine, National University of Singapore, Singapore; 5 Center for Tropical Medicine and Global Health, Nuffield Department of Medicine, University of Oxford, Oxford, UK; 6 Mahidol-Oxford Research Unit, Mahidol University, Bangkok, Thailand

**Keywords:** clinical trial, medical ethics, patient participation, systematic review

## Abstract

**Abstract:**

**Objectives:**

Randomised trials for the management of drug-resistant infections are challenging to conduct as target patient populations often lack decision-making capacity, and enrolment windows are typically short. Improving informed consent and risk communication in these trials is especially crucial for protecting patient interests and maximising trial efficiency. This study aimed to understand challenges in risk communication and informed consent in antimicrobial clinical trials.

**Design:**

Scoping review.

**Data sources:**

Searches were conducted in Embase, Medline, CINAHL and Web of Science Core for peer-reviewed English articles that were published from January 2000 to April 2023.

**Eligibility criteria:**

Included articles were empirical studies or expert opinions that sought experts’, patients’ or representatives’ opinions on informed consent in the context of clinical trials involving antibiotic/anti-infective agents.

**Data extraction and synthesis:**

Abstract screening, full-text review, data extraction and evidence rating were performed by two independent reviewers. Extracted data were summarised and reported qualitatively based on common themes. A total of 2330 records were retrieved, and 29 articles were included in the review.

**Results:**

Half of the articles involving medical experts and one-third involving patients and representatives reported that full comprehension by patients and representatives was challenging or not achievable. Healthcare providers and consent takers were crucial for the quality of informed consent. The level of trust consent givers placed on healthcare providers had a critical influence on the consent rate. Emotional distress was pervasive among patients/representatives.

**Conclusion:**

The findings indicate that strengthening consent takers’ communication skills in providing emotional support to patients and their representatives may improve informed consent. More research is needed to understand informed consent in low-income and middle-income and non-English-speaking countries.

**STRENGTHS AND LIMITATIONS OF THIS STUDY**

- This study includes views from experts and patients or representatives on informed consent.
- This study advances the understanding of challenges in informed consent in antimicrobial trials.
- The main limitation is that this study predominantly focuses on bacterial infections and thus has limited generalisability to other types of trials.

## Introduction

 Expensive and inefficient randomised trials for novel antibiotics and diagnostics are key factors contributing to the ‘valley of death’ for research and innovation in this field.^1^ This leads to delays in regulatory approvals for these life-saving drugs and deters pharmaceutical companies from investing in antimicrobial drug discovery.^2 3^ One contributing hurdle to inefficiency in these trials is low consent rates coupled with poor quality of informed consent.^4^,^7^ Poor quality of informed consent can harm the public’s trust in healthcare and medicine. Slow recruitment in clinical trials threatens internal validity by increasing the risk of confounding factors, differential attrition and operational drift, while it compromises generalisability by potentially altering the target population, reducing temporal relevance and introducing selection bias.^8 9^

Informed consent involves ‘voluntary authorisation, by a patient or research subject, with full comprehension of the risks involved’^10^ and is one fundamental ethical requirement for human subject research. Risk and uncertainty exist when information is incomplete, and our knowledge of the negative outcomes, benefits or other aspects of a medical treatment is limited during the informed consent procedure.^11^,^13^ In most medical research, risk usually refers to the possibility of having undesirable outcomes such as adverse effects. Poor communication of the trial information is one of the main reasons for the ineffective informed consent.^8^

Treatment strategy trials for multidrug-resistant infections hold unique challenges for informed consent. These challenges include strict enrolment criteria, limited time frame for enrolment, and target patient populations not having decision-making capacity for consent due to underlying severe infections. Specifically, the window for recruitment and consent is often narrow as the antibiotics under evaluation need to be administered as quickly as possible to control infections.

These challenges are exacerbated by other pervasive reasons behind poor understanding of informed consent forms and low consent rates for other types of clinical trials. Several studies found that information sheets, including templates provided by institutional research boards, are difficult to read,^14 15^ have great variability or insufficient explanation when stating risks and/or benefits^16 17^ and might not encourage decisions that meet recommendations such as the International Patient Decision Aids Standards instrument.^6^ The issue might be exacerbated by language and literacy barriers, especially those in low-income to middle-income countries.^18^ Second, doctor–patient communication is often inadequate in explaining complex concepts such as randomisation, placebo and priority given to patient well-being.^4 19^ While several strategies such as improving doctor–patient communication and relationships have been implemented to optimise recruitment in clinical trials, there is a lack of evidence-based strategy.^8^ Despite the introduction of ‘good clinical practice’ guidelines by the WHO,^5 20^ systematic reviews show that participants’ understanding of clinical trials, especially risk and side effects, had no substantial improvement over the past two decades.

There is a need for evidence-based strategies which balance individual patient autonomy and broader societal justice derived from successfully completed clinical trials. The current review aimed to understand the challenges in informed consent in the context of antimicrobial trials, by focusing on issues around risk communication, including patients’ concerns about the risk and uncertainty from experts’ and consent givers’ perspectives. We sourced both empirical studies that address patients’ perspectives and articles that present domain experts’ views. The specific objectives are to ascertain: (1) experts’ views and recommendations on risk communication; (2) patients’ or representatives’ concerns around risk and uncertainty when deciding on participation and (3) how communication of trial information and other factors could influence consent in the context of antimicrobial clinical trials.

## Methods

### Search strategy

We conducted searches in the following databases: Embase via Elsevier, Medline via Elsevier, PsycINFO via Ovid, CINAHL via EBSCOhost and Web of Science Core. The initial searches were conducted on 26 December 2022, and update searches were conducted on 26 April 2023. The search strategy aimed to locate peer-reviewed articles published in the English language from January 2000 for relevance and recency considerations in relation to treatment approaches and regulatory aspects. The details about the searches and full-search strategies are found in online supplemental material. All results were collated using both the SR-accelerator^21^ and EndNote.

### Data selection

The inclusion criteria were (1) in the context of clinical trials involving antibiotic/anti-infective agents; (2) empirical studies (eg, qualitative or quantitative) or an expert opinion guideline (experts defined in this review included health professionals, academics or researchers, research staff and regulators) and (3) addressed one or more of the following topics: patients’ willingness to participate in trials; risk and benefit considerations when participating in trials; content of informed consent; ethical issues relating to informed consent. The exclusion criteria were (1) studies that tested the efficacy or safety of a drug; (2) focused on antibiotic prescription in healthcare settings or (3) articles that emphasised cases (eg, vaccines, parasites, HIV or tuberculosis) that have more unique treatment approaches and regulatory considerations, and patients are typically less acutely unwell or a decision for treatment was less urgent. Title and abstract screening and full-text screening were performed by two reviewers (YS and AS-RP). Discrepancies in selecting the final included studies were resolved by consensus or a third reviewer (YM). Data selection was performed using SR accelerator and COVIDENCE.^22^

The quality of evidence from each shortlisted study was rated by two reviewers (YS and JEY) based on the modified Oxford Centre for Evidence-Based Medicine (OCEBM) levels of evidence. Level 1 referred to the highest level of quality (including randomised controlled trials with proper power) while level 5 referred to the lowest level of evidence (including case reports and opinions).^23^

### Data extraction

Data extracted included the country/countries where the study was conducted, the type of clinical trial, and the target patient population. Data extracted for empirical studies also included study sample details (sample size and sample characteristics), methods (survey, interview and focus groups), and results and themes relating to informed consent. Data extracted from experts’ articles included opinions and statements in relation to consent. Initial data extraction was performed by two independent reviewers (any two of JEY, AS-RP and YS). The aggregated data were then reviewed and revised by all reviewers (JEY, AS-RP and YS). The extracted qualitative data were synthesised in a narrative format and categorised based on common themes by YS and were revised by JEY. All authors reviewed the final themes.

### Patient and public involvement

None.

## Results

**Figure 1:**
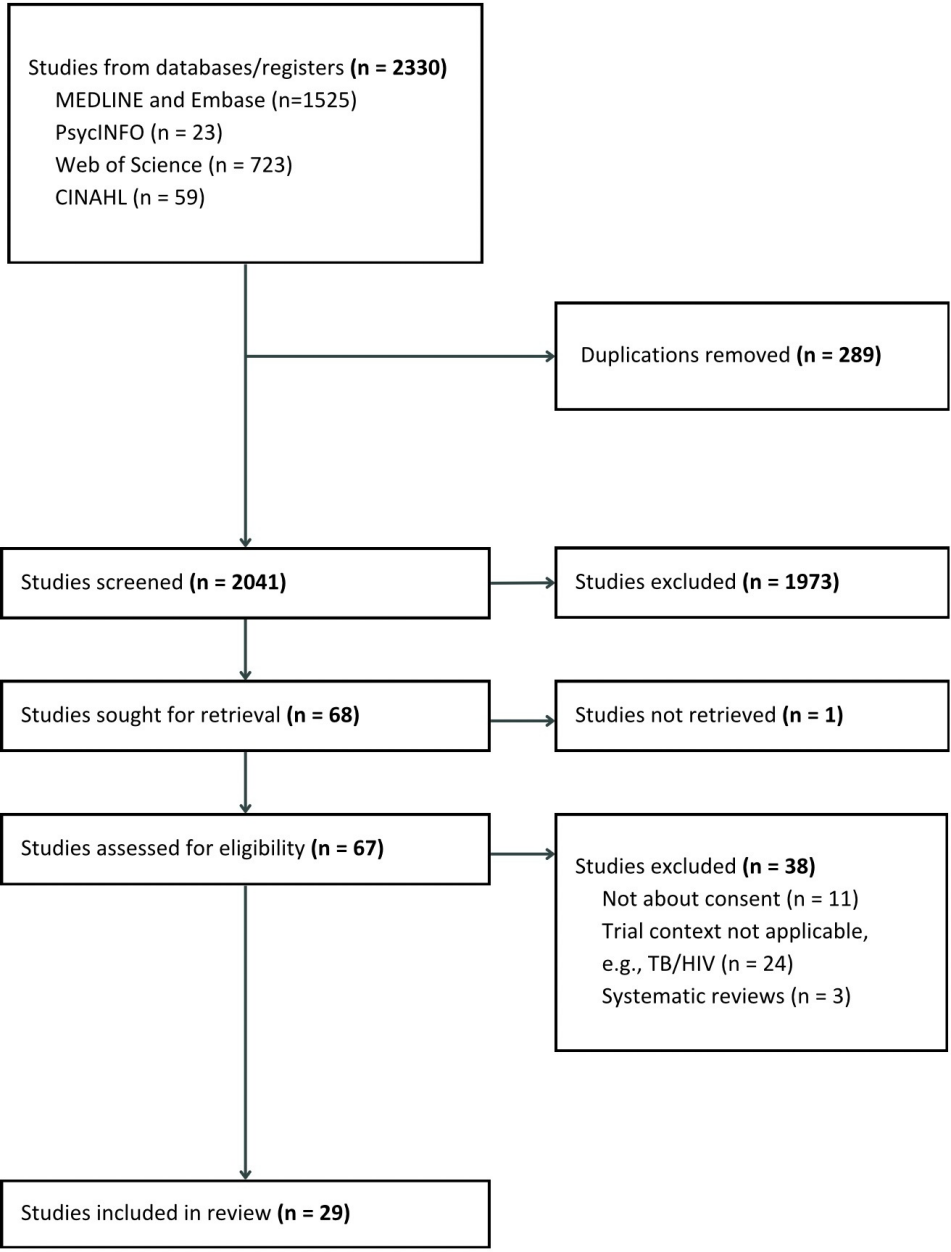
PRISMA flow chart of evidence selection. PRISMA, Preferred Reporting Items for Systematic Reviews and Meta-Analyses; TB, tuberculosis.

A total of 2041 unique records were screened and assessed by two independent reviewers. A total of 29 articles were selected for data extraction. These included 14 experts’ opinions, 11 studies that focused on the views of patients or representatives and 4 included both expert and patient responses (see figure 1). Three, 1, 11 and 14 articles were from OCEBM levels 1, 3, 4 and 5 evidence, respectively.

Among the 18 articles based on experts’ views (12 articles by individual experts and 6 articles summarising aggregated experts’ views), the vast majority of the experts were doctors or medical researchers in English-speaking high-income countries such as the USA, the UK, Canada and Australia (17/18, 94%) (table 1). Three articles focused on informed consent for minors, two for pregnant women, one for older adults and one for participants in developing countries. Among the 15 articles based on patients’ and representatives’ views, 5 focused on minors, 2 on pregnant women and 1 on older adults (see table 2).

**Table 1:** Characteristics of included papers synthesising expert views

Citation	Trial-related context	Country of the trial/context	Type	Expert background	Level of evidence
^37^	Prophylactic antibiotics for neurosurgical procedure including clinical trials	USA	Opinion	Doctor/researcher	5
^28^	Trovafloxacine for meningitis in child trialTarget patient: Minors	Nigeria-Kano	Opinion	Researcher in sociology	5
^24^	Phase IV clinical trials Target patient: Pregnant women	USA	Opinion	Doctor/researcher	5
^26^	The Closed or Open after Laparotomy (NCT03163095) Study (clinical trial for severe complicated intra-abdominal sepsis)	Canada	Opinion	Doctor/researcher	5
^31^	Pragmatic trials for pneumonia	USA	Opinion	Doctor/researcher	5
^36^	Clinical trials for COVID-19 treatments and vaccines	International	Opinion	Doctor/researcher	5
^47^	Pharmacogenetics to Avoid Loss of Hearing trial (ISRCTN13704894) Target patient: Minors Consent giver: Parents	UK	Opinion	Doctor/researcher	5
^32^	Phase 1/2 clinical trials	–	Opinion	Pharmacological researchers	5
^27^	–	UK	Opinion	Doctor/researcher	5
^33^	Evaluating Diuretics in Normal Care Study (ISRCTN46635087)Cluster randomised trials of hypertension prescribing policyDiscussed consent mode: opt-in/out	UK	Opinion	Doctor/researcher	5
^51^	Trials among stroke patients	USA	Opinion	Doctor/researcher	5
^30^	–	–	Opinion	Veterinarian	5
^29^	Clinical trials for Hospital-Acquired/Ventilator-Associated Bacterial Pneumonia	USA	Meetings involving doctors and research staff in 2013	An expert team of various stakeholders including academic scientists, clinicians, regulators, trial monitors and coordinators, and patient and industry representatives	5
^34^	Clinical trials for COVID-19 treatments and vaccinesTarget patient: Pregnant women	USA	A public meeting involving doctors and research staff in 2021	Stakeholder categories including academia, industry, governmental agencies and patient advocacy groups	5
^38^	Noninferiority treatment trial for healthcare associated pneumonia Discussed consent mode: Advanced consent	USA	Interviews and meetings involving health professionals, research staff and IRB members	10 IRB representatives; 7 investigators; 5 study coordinators	4
^25^	Noninferiority treatment trial for healthcare associated pneumonia Discussed consent mode: Advanced consent	USA	Interviews and meetings involving doctors, research staff and IRB members during 2017–2018	10 IRB representatives; 7 investigators; 5 study coordinators	4
^43^	CONservative TReatment of Appendicitis in Children a randomised controlled Trial (ISRCTN15830435) Target patient: Minors Consent giver: Parents	UK	Interviews with doctors during 2017–2018	35 health professionals(25 surgeons, 7 research nurses, 3 ward nurses)	4
^35^	Probiotics for Antibiotic Associated Diarrhoea study (ISRCTN 7954844)Target patient: Older adults in care homesDiscussed consent mode: Advanced consent	UK	Interviews with doctors and staff in 2013/2014	19 care home staff; 10 general practioners	4

IRBinstitutional review board

**Table 2:** Characteristics of included papers synthesising views of patients and representatives

Citation	Trial-related context	Country of the context	Study year	Method	Participants characteristics	Level of evidence
^35^	Probiotics for Antibiotic Associated Diarrhoea study (ISRCTN 7954844)Target patient: older adults in care homesConsent mode: Advanced consent	UK	2013–2014	Interview	14 residents in age cares14 relatives (4 partners, 10 children)	4
^42^	Overview of the Role of Antibiotics in Curtailing Labour and Early delivery -Antibiotics for Preterm, Prelabor Rupture of Membranes trial (ISRCTN53994660)Target patient: Pregnant women	UK	–	Interview	20 patients	4
^44^	Overview of the Role of Antibiotics in Curtailing Labour and Early delivery -Antibiotics for Preterm, Prelabor Rupture of Membranes trial (ISRCTN53994660)Target patients: Pregnant women	UK	–	Interview	38 patients (age range: 28–59)	4
^38^	Noninferiority treatment trial for healthcare associated pneumoniaDiscussed consent mode: Advanced consent	USA	2016	Interview	18 patients (22% male, age range: 29–75, 10 had tertiary education)12 caregivers (33% male; 4 had tertiary education)	4
^25^	Noninferiority treatment trial for healthcare associated pneumoniaDiscussed consent mode: Advanced consent	USA	2016–2017	Delphi method including semistructured telephone interview and surveys	Interview study sample same as^38^	4
^43^	CONservative TReatment of Appendicitis in Children a randomised controlled Trial (ISRCTN15830435)Target patients: minorsConsent giver: parents	UK	2017–2018	Interview	28 families (15 with mothers only, 7 with fathers only, 6 with both parents); and14 children completed interviews	4
^39^	Initial goal is antibacterial drug development paediatric trials; later expanded to any paediatric trials (including antibiotics)Target patient: minorsConsent giver: parents	USA	2015	Interview	24 parents (19 consented trial participation, 5 declined trial participation)	4
^46^	Prevention of Recurrent Urinary Tract Infection in Children with Vesicoureteric Reflux and Normal Renal Tracts study (ACTRN12608000470392)Target patients: minorsConsent giver: parents	Australia	–	Secondary data analysis mainly	1109 parents(412 consented to clinical trial participation 697 declined but gave reasons)	4
^67^	The High Flow Nasal Cannulae as Primary Support in the Treatment of Early Respiratory Distress trial (ACTRN12613000303741)Target patients: minorsConsent giver: parentsConsent mode: prospective and retrospective consent	Australia	2013 (era 1)2014 (era 2)	Secondary data analysis	220 eligible babies in era 1 (111 with consent: 53% male, mean gestational age=31.1 weeks)209 eligible babies in era 2 (171 with consent: 56% male, mean gestational age 31.1 weeks)	3
^41^	Single site, double-masked, randomised, placebo-controlled trial to evaluate intravenous doxycycline for rheumatoid arthritis	USA	–	Survey	30 baseline patients (20% males, mean age=44.9, median of 12.5 years of education)26 follow-up patients	4
^40^	Treatment of acute uncomplicated appendicitis comparing surgery to conservative management with antibioticsTarget patient: minorsConsent giver: parents	Singapore	2017–2018	Survey	113 patients’ parents(patients: 59.3% male, mean age=9.7; parents: 33.6% father, mean age=41.2, 39.8% had tertiary education)	4
^45^	Hypothetic randomised controlled antibiotic trials	UK	–	Experiment via online survey	1067 participants(48.80% male, age range=14.9% 65–75, 16.2% 55–64, 18.7% 45–54, 17.2% 35–44, 18.7% 25–34, 14.2% 16–24; 52.1% had tertiary education)	1
^49^	Comparison of Outcomes of antibiotic Drugs and Appendectomy trial (NCT02800785)(pragmatic, nonblinded, noninferiority, multicenter RCT comparing antibiotics and surgery for acute appendicitis)	USA	2016–2020	Experiment	4627 patients(55% male, age: 39% 18–29, 26% 30–39, 16% 40–49, 10% 50–59, 6% 60–69, 2% above 70;3111 patients declined randomisation)	1
^48^	Hypothetic RCT antibiotic trials	UK	–	Experiment via online survey	443 participants(18.30% male, mean age=25.5, 47% had had tertiary education)	1
^50^	Oral ciprofloxacin with nebulised colistin vs intravenous anti-pseudomonal antibiotics for Pseudomonas aeruginosa infection Target patient: patients with cystic fibrosis	UK	2006	Survey	106 consumers(42% male, 56% respondents were parents)	4

### Achieving informed consent is challenging

A frequent concern among experts was that true informed consent with full comprehension by patients and representatives was challenging or not achievable^24^,^31^ (table 3). One reason was that because clinical trials are meant to establish evidence or explore uncertainties for the interventions they are testing, specific risks may not be clearly known at the time of research.^2427 32^,^34^ Other reasons included patients and representatives being unable to fully understand the research,^25 31 35^ due to a lack of health literacy, complexity of research terms, and cultural and language barriers. While improving patients’ understanding^28 29 36 37^ was frequently recommended for improving informed consent, experts were also concerned that patients might have cognitive impairment or declined cognitive capacity in acute illness, who might be deemed to have decision-making capacity but unable to fully comprehend the complexities of the proposed research.^26 27 29 35^

**Table 3:** Summary of main findings

Experts	Citations	Patients and representative	Citations
Informed consent and patient understanding			
True informed consent can be challenging		Patients and representative can have misunderstandings	
Risk and uncertainty are the nature of the research; risks may not be clearly known at the time of research	2427 30 32 34	Lack the understanding or misunderstanding of risk; or believe in minimal or no risk; believe risks should have been known already	^41 42 44 45^
Patients or representatives may not fully understand or misunderstand the research/risk; not pay attention or quickly forget the information	^25 28 31 35^	Lack the understanding or misunderstanding of research design	39 43
Patients may have impairment or do not have the capacity of decision-making	^26 27 29 35^	Inaccurate/overoptimistic/overestimate of benefit	^41^
Cultural and language barriers in developing countries may negatively impact comprehension	^28^		
(Elderly) Participants may quickly forget the purpose of the study	^35^		
How much information should be given is not clear cut	^37^	Knowing information about the research and trial is important for patients and representatives	2538 40
Improving patient understanding, and patient education are recommended	^28 29 36 37^		
Doctors/research staff are critical			
Doctors/research staff have the responsibility to explain risks, including antimicrobial-resistant risk in antibiotic trials	^24 27 33 37^	Patients and representatives are influenced by:	
Doctors/staff’s own preference and understanding may result in biased explanation or wording when communicating with patients	^25 43^	Doctors' attitudes and opinion, and how doctors frame risks	^39 40 42 43 46^
Doctors/staff should provide counselling to patients; discussion with patients such as exploration of options	^24 27 28 37 43 47^	Counselling and discussion with doctors and staff	^39 43^
Coercive decisions during informed consent may happen	^27 28^	Trust in/preferences of staff or doctors; believe that staff or doctors have their best interest	^39 41 42 44 46^
Staff/doctor training and improve communication/language of risk communication are recommended	^29 36 43^	Friendliness and empathy of staff	^39 40 42^
Senior/more experienced staff have better consent rate	^35^		
Information leaflets and consent forms			
Staff indicated that representatives may want simple explanations and can be put off by the lengthy information sheet	^35^	Participants may not interpret the information in consent forms as what is intended to be convened	^44^
Consent forms should provide balanced information about alternatives	^25^	Framing and format of consent form may influence risk perception when participants have sufficient time to read information; but may not influence consent	^45 48 49^
		Some patient information leaflets poorly inform people about risk	^45^
Patients’ considerations in consenting			
Factors specific to trial properties and outcomes			
Altruism	^32 34^	Benefit other patients like them and benefit science and research	3540 42 44 50
Risk–benefit considerations including long-term ones; uncertainty around the treatment	^31 32 36^	Patient benefits from the treatment, hope	^35 39 41 42 44^
		Safety/minimal risk, side effects and health risk to patients and/or their unborn child	^35 39 40 42 44 49^
Logistics/time/convenience/ transport	^27 32 36^	Logistics/time/convenience/transport	^39 46 50^
Financial incentives/barriers	^32^	Reimbursement/incentives; costs related to the treatment	^39 40 49^
Social interaction with others during trial participation	^32^	Disruption to social life	^39^
		Interest	^35 49^
		Believe to have better medical care via trial participation	^41^
		Concerned about blinding	^42^
		Privacy and confidentiality	^49^
Other key factors/concerns			
Trust in medicine	^28 36^	Trust in regulation, system or authorities	^39 40 42 44^
Partnership, patients’ knowledge and contribution are acknowledged	^32^	Trust in research and researchers (eg, researchers will aim for more benefits and less risks for patients)	^40 44^
Reliable information and source of information	^34 36^	Family or friends’ recommendations	^41^
		Having preferences on treatment options	^40 46 49 50^
		Autonomy	^40 49^
		Having the right to withdraw	^38 40^
		Sociodemographic factors (eg, education, age of patients, language spoken at home)	^40 46^
Consent procedure			
Issues related to time			
Time constraint in regular doctor consult session and variation in patient background	^31^	Time pressure; limited processing of information, rely on common sense/heuristics	42 44
Should allow sufficient time for patients to understand information and make decisions	^27 28^	Some may make decisions with little consideration or straightway	^43 44^
		Timing of approaching for recruitment is important	^39^
Health professionals and staff may be concerned about worrying families about treatment risks	^43^	Emotional distress, anxiety, fear, worry	38 4042
Consent procedures especially complex ones take time and increase workload	^29 31 33 35 38^		
IRB complications and issues impose challenges	^29 31^		
Consent mode			
Consider advanced consent and early enrolment	^29 35 38^	No concerns over advanced consent and early enrolment	^35 38^
Waiver or deferred consent	^26 31^	Retrospective consent may increase consent rate	^67^
The usual prior consent can be impractical or difficult, especially in urgent situations	^26 27 29 51^		
The legally authorised representative should be communicated in any trial participation conversations	^29 38^		
Opt-in/opt-out recruitment	^31 33^		
Use eConsent	^32^		
Not all situations can omit consent process	^47^		

On the other hand, patients and representatives valued being well-informed and receiving information about the research.^2535 38^,^40^ However, recurrent themes included the difficulty, lack of or misunderstanding of research and trial designs, especially randomisation and blinding.^39^,^43^ Patients had an inaccurate understanding and underestimated the risk of the research.^41 42 44 45^ Patients believed that there was minimal or even no risk involved in the research^44^ while overestimating the benefit or being overoptimistic about the treatment.^41^

### Doctors and research staff are critical for the success and quality of consent

The experts generally agreed that doctors and research staff hold the responsibility to explain risks to patients.^24 27 33 37^ However, doctors’ and research staff’s own preferences, understanding, and experiences might influence risk communication with patients and patients’ consent.^25 35 43^ Corneli *et al*^25^ reported that the doctors and research staff might have misconceptions about terms like non-inferiority, and their misunderstanding could negatively impact their risk communication with patients. Similarly, staff or doctors-related factors were the most commonly raised^39^,^4446^ by patients and representatives. Those factors included trust in doctors and research staff,^39 41 42 44 46^ doctors’ attitudes and opinions and how they frame risks during the communication,^3941^,^44 46^ and friendliness^40^ and sympathy^39 42^ from the staff. Furthermore, the need for counselling or discussion between patients and representatives and doctors and staff, including exploring alternative options^39 43^ was proposed by patients, representatives and experts.^24 27 28 43 47^ Providing training to doctors and staff^29 36 43^ was recommended for improving informed consent.

### Consent forms

Several articles mentioned informed consent forms having either too much information, insufficient details for participants to understand the research or being prone to misinterpretation by participants.^35 44 45 48^ Three articles investigated the effect of the format and framing of information sheets on participants’ perceptions or consent.^45 48 49^ The framing of the side effects might influence risk perceptions when participants spent adequate time reading the information but did not appear to influence consent or perceived research credibility.^45^

### Patients’ concerns centred around risks and benefits to individual and wider population

Experts recognised a range of factors that influence patients' decision to provide informed consent, especially those relating to trial properties and outcomes such as the study’s risk and benefit,^31 32^ altruism,^31 32^ convenience (eg, logistics, flexibility in time),^27 36^ financial hurdles^32^ and social interaction with others and partnership (eg, patients’ expertise, trust and contribution are acknowledged) during the trial participation.^32^ Similar factors were mentioned by patients and representatives, including health-related risk and outcomes,^35 39 40 42 44 49^ perceived benefit to the patient’s health condition and hope,^35 39 41 42 44^ altruism (eg, benefiting science and medical research, and other patients),^3540^,^42 44 50^ logistics and opportunity cost,^39 46^ incentives and cost incurred due to complications,^40 49^ and disruption to social lives.^39^ Patients and representatives were also motivated by their interest in the study^35 49^ and the belief that they might receive better care^41^ through trial participation.

Both experts and patients also indicated trust as an important factor, including patients’ trust in medicine,^28 36^ the system and government regulation,^39 40 42 44^ and science and medical research.^40 44^ Patients’ rights to withdrawal, autonomy (eg, being able to make a choice or act based on their will) and having had a decision or preference for a specific treatment option were also frequently mentioned.^40 46 49 50^

### Consent procedures can be time-constrained and distressing

Experts expressed that the consent taking procedures, especially complex ones, can be laborious and increase the workload of healthcare professionals.^29 31 33 35 38^ While experts recommended allowing more time for consent givers to make decisions,^27 28 43^ time-related issues such as time pressure were experienced by both experts and consent givers.^42^,^44^ Recruiting doctors might face the challenge of time constraints during the usual doctor consultation.^31^ Meanwhile, consent givers reported that they relied on common sense and heuristics during decision-making^44^ and might have had little consideration during the process.^43 44^

It was also observed that negative emotions, especially emotional distress, during the decision process among patients and representatives were reported in almost all the primary research studies.^38^,^44^ Anxiety, fear, and worry were the common emotions expressed or shown by patients and representatives. Relating to the consent takers factors above, patients appreciate empathy from recruiting staff.^39 42^

### Alternatives to conventional consenting process

Experts expressed concern that conventional informed consent after infection onset can be impractical.^26 27 29 51^ Some experts suggested the implementation of advanced consent and early enrolment (consent and enrolment before a patient becomes eligible for a study) prior to infection onset.^29 35 38^ Patients and relatives also expressed no major concerns about early recruitment/enrolment or advanced consent.^35 38^

## Discussion

The current review explored challenges in informed consent by focusing on risk communication, including patients’ concerns about risk and uncertainty, in the context of antimicrobial trials. One key finding in our review was that achieving true informed consent can be challenging. Doctors and research staff were suggested to be the most essential in the informed consent and risk communication process. Trust in doctors and staff, medical research, and the healthcare and regulatory systems were key influences during consent givers’ decision-making. Lastly, there was pervasive emotional distress among patients and representatives during the consent procedure.

The finding that true informed consent might not be achieved, either due to the lack of understanding or the lack of capacity from patients and representatives, aligned with previous systematic reviews that consent givers’ misunderstanding of clinical trials was one of the main issues in informed consent.^5 20^ Given that clinical research is difficult to explain, patients’ trust in doctors and research becomes critical for informed consent. The role of trust in patient decisions is also discussed in the previous literature.^4 52^ Believing that doctors and staff have their best interests and that safety is ensured via strict regulation reassures consent givers that any risks or negative consequences will be managed and minimised. However, trust could also be a double-edged blade, especially when consent givers do not have an accurate understanding of the research. Doctors and research staff may consciously or unconsciously express their own preferences and biases when communicating with consent givers and sometimes may even have misconceptions about the research. These, in turn, influence consent givers’ understanding and decisions. Consent givers might also overly rely on trust rather than engaging in understanding the research. The experience of adverse effects that were not expected by patients due to misunderstanding can result in substantial damage to their trust in medicine.^28 44^

Furthermore, we observed that consent givers, including patients and family members, expressed anxiety, fear, worry, and feeling overwhelmed during the decision process. This is in line with the observation by a previous study that found that anxiety associated with these high-stakes interventions may impact patients’ ability to understand the documents and make informed decisions about participation in the trial.^15^ Anxiety and fear can influence risk and benefit perceptions, thus influencing informed decision.^53 54^ Managing consent givers’ negative emotions and showing empathy and sensitivity by staff can be important during the informed consent procedure.

Our review did not find evidence that informed consent forms played a crucial role in consent for antimicrobial clinical trials. In fact, many participants might spend little time reading the information sheets in hypothetical clinical trials.^49^ Consent givers in real trial settings might feel having little time to process the given information, and thus may largely rely on heuristics.^55^,^57^ Although it has been recommended that sufficient time should be allowed for consent givers to understand the information and make decisions,^27 28 43^ time constraints can still be challenging, especially in trials with narrow recruitment windows. An alternative solution is allowing advanced consent and early enrolment (ie, before patients become eligible), to address issues including patients having limited decision time or lack of decision capacity, which were found acceptable by both experts and patients or their representatives.

We found a lack of research on informed consent in antimicrobial resistance trials in low-income to middle-income countries. This contrasts with a review by the US Food and Drug Administration, which included 42 phase 3 antibiotic trials that showed just 16.7% of participants were from the USA.^58^ A recent systematic review found that the consent rate in low-income to middle-income countries was significantly higher than in high-income countries.^59^ However, the quality of the informed consent might be questionable as language and cultural barriers in developing countries might exacerbate the comprehension issues in informed consent.^60^,^63^ Participants’ consent in developing countries might also be influenced by unique factors such as social influence,^60^ free medical care and opportunities to gain knowledge and skills during the trial participation.^61 62^ Meanwhile, significant disparities exist where middle-income and lower-middle-income countries have limited access to healthcare including antibodies.^64^ Risks and benefits of trials and participants’ motivations to consent in middle-income and lower-middle-income countries encompass a unique set of ethical challenges.^65^ It is critical to understand informed consent from participants in low-income to middle-income countries.

Several limitations of this review should be noted. First, we included articles which predominantly focused on bacterial infections. However, our findings may be extrapolated to other medical conditions and clinical trials which are time-sensitive. Second, we focused on risk and uncertainty communication during informed consent. Future research may have broader investigations on other factors that may influence informed consent. Furthermore, challenges in recruitment and issues of trial validity go beyond those in risk communication, comprehension, and acceptance of trial participation. The extent to which a trial is inclusive in reaching patients from diverse backgrounds also influences the trial recruitment and generalisability of the trial results. Inclusiveness and diversity have been increasingly emphasised by both scientific communities and regulatory bodies.^66^ Future research should have a more in-depth understanding of the interplay between consent, inclusiveness, and diversity in trial conduct.

Finally, the articles in the current review are exclusive academic articles and have been more focused on issues relating to consent givers. Successful recruitment, effective risk communications and high-quality conduct of trials can depend on investigators’ ability to conduct trials and the availability of the research staff to invest in the time to facilitate consent. Future research should also include challenges relating to trial investigators and regulators (eg, institutional review boards) and review literature beyond traditional academic publications.

In conclusion, our review found that difficulty in achieving full informed consent and adequate comprehension among patients and representatives, exacerbated by a narrow consent window, are the major challenges in antimicrobial trials. Improving professionality, communication skills and empathy among doctors and staff may improve consent quality, reduce negative emotions associated with the consent procedure, and promote trust building. Table 4 summarises the main recommendations for improving informed consent and consent rate based on the articles and current review. Meanwhile, more research and empirical evidence are needed to develop more systematic and effective guidance for those recommendations. The current review also highlights the knowledge gap in developing countries and non-English-speaking populations and calls for more research in under-researched populations.

**Table 4:** Recommendations for improving informed consent and consent rate

Challenges	Recommendations
Risk (mis)communication	Provide training to recruiting doctors and consent takers to improve communication of trial information and better manage patients’ and representatives’ expectations of risk
Emotional distress of patients and representatives	Provide training to recruiting doctors and consent takers to improve interpersonal skills to be more sensitive to patients’ circumstances and approach patients and representatives at an appropriate time. be more empathetic and manage negative emotions of patients and representatives.
Refusals due to trial-related barriers	Involve patients and representatives in study design including informed consent process. Identify local cultural barriers of consent among patients and representatives; address the manageable barriers (eg, logistics, cost, social isolation) accordingly.
Refusals due to misperception of clinical trials	Public engagement to increase awareness and trust in clinical trials.

## supplementary material

10.1136/bmjopen-2023-082096 online supplemental file 1

## Data Availability

Data are available on reasonable request.

## References

[1] Chorzelski S, Grosch B, Rentmeister H Enhancing Research and Development of Antibiotics in Science and Industry [Internet].

[2] Wagenlehner FM, Gasink LB, McGovern PC (2024). Cefepime-Taniborbactam in Complicated Urinary Tract Infection. N Engl J Med.

[3] Eckburg PB, Muir L, Critchley IA (2022). Oral Tebipenem Pivoxil Hydrobromide in Complicated Urinary Tract Infection. N Engl J Med.

[4] Abraham NS, Young JM, Solomon MJ (2006). A systematic review of reasons for nonentry of eligible patients into surgical randomized controlled trials. Surgery.

[5] Pietrzykowski T, Smilowska K (2021). The reality of informed consent: empirical studies on patient comprehension-systematic review. Trials.

[6] Brehaut JC, Carroll K, Elwyn G (2015). Elements of informed consent and decision quality were poorly correlated in informed consent documents. J Clin Epidemiol.

[7] Montalvo W, Larson E (2014). Participant comprehension of research for which they volunteer: a systematic review. J Nurs Scholarsh.

[8] Fletcher B, Gheorghe A, Moore D (2012). Improving the recruitment activity of clinicians in randomised controlled trials: a systematic review. BMJ Open.

[9] Caldwell PHY, Hamilton S, Tan A (2010). Strategies for increasing recruitment to randomised controlled trials: systematic review. PLoS Med.

[10] National Library of Medicine (1973). nformed Consent - MeSH - NCBI.

[11] Tversky A, Fox CR (1995). Weighing risk and uncertainty. Psychol Rev.

[12] Smithson M (2010). Understanding uncertainty. Dealing with uncertainties in policing serious crime.

[13] Kalke K, Studd H, Scherr CL (2021). The communication of uncertainty in health: A scoping review. Pat Educ Couns.

[14] Paasche-Orlow MK, Taylor HA, Brancati FL (2003). Readability Standards for Informed-Consent Forms as Compared with Actual Readability. N Engl J Med.

[15] Nathe JM, Krakow EF (2019). The Challenges of Informed Consent in High-Stakes, Randomized Oncology Trials: A Systematic Review. MDM Policy Pract.

[16] Kahrass H, Bossert S, Schürmann C (2021). Details of risk-benefit communication in informed consent documents for phase I/II trials. Clin Trials.

[17] Kirby N, Shepherd V, Howick J (2020). Nocebo effects and participant information leaflets: evaluating information provided on adverse effects in UK clinical trials. Trials.

[18] Tamariz L, Palacio A, Robert M (2013). Improving the informed consent process for research subjects with low literacy: a systematic review. J Gen Intern Med.

[19] Mills EJ, Seely D, Rachlis B (2006). Barriers to participation in clinical trials of cancer: a meta-analysis and systematic review of patient-reported factors. Lancet Oncol.

[20] Tam NT, Huy NT, Thoa LTB (2015). Participants’ understanding of informed consent in clinical trials over three decades: systematic review and meta-analysis. Bull World Health Organ.

[21] Clark J, Glasziou P, Del Mar C (2020). A full systematic review was completed in 2 weeks using automation tools: a case study. J Clin Epidemiol.

[22] Veritas Health Innovation Covidence systematic review software, Melbourne, Australia.

[23] American Medical Association (2023). JAMA Network Open Instructions for Authors - Ratings of the quality of the evidence. https://jamanetwork.com/journals/jama/pages/instructions-for-authors.

[24] Briggs GG, Polifka JE, Wisner KL (2015). Should pregnant women be included in phase IV clinical drug trials?. Am J Obstet Gynecol.

[25] Corneli A, Calvert SB, Powers JH (2020). Consensus on Language for Advance Informed Consent in Health Care-Associated Pneumonia Clinical Trials Using a Delphi Process. JAMA Netw Open.

[26] Doig CJ, Page SA, McKee JL (2019). Ethical considerations in conducting surgical research in severe complicated intra-abdominal sepsis. *World J Emerg Surg*.

[27] Green JS, Pace N (2006). Ethics of clinical trials. Anaesth & Intensive Care Med.

[28] Jegede AS (2009). Understanding informed consent for participation in international health research. Dev World Bioeth.

[29] Knirsch C, Alemayehu D, Botgros R (2016). Improving Conduct and Feasibility of Clinical Trials to Evaluate Antibacterial Drugs to Treat Hospital-Acquired Bacterial Pneumonia and Ventilator-Associated Bacterial Pneumonia: Recommendations of the Clinical Trials Transformation Initiative Antibacterial Drug Development Project Team. Clin Infect Dis.

[30] Menache A (2003). The Era of Valid Informed Consent Informed Consent. Med & L.

[31] Monach PA, Branch-Elliman W (2021). Reconsidering minimal risk’ to expand the repertoire of trials with waiver of informed consent for research. BMJ Open.

[32] Iersel T van, Courville J, Doorne C van (2022). The Patient Motivation Pyramid and Patient-Centricity in Early Clinical Development. Curr Rev Clin Exp Pharmacol.

[33] Rogers A, Craig G, Flynn A (2020). Cluster randomised trials of prescribing policy: an ethical approach to generating drug safety evidence? A discussion of the ethical application of a new research method. Trials.

[34] Sewell CA, Sheehan SM, Gill MS (2022). Scientific, ethical, and legal considerations for the inclusion of pregnant people in clinical trials. Am J Obstet Gynecol.

[35] Wood F, Prout H, Bayer A (2013). Consent, including advanced consent, of older adults to research in care homes: a qualitative study of stakeholders’ views in South Wales. Trials.

[36] Russell JA, Walley KR, Kalil AC (2022). The Potential for Increasing Risk of Consent Refusal in COVID-19 Trials: Considering Underlying Reasons and Responses. Ann Am Thorac Soc.

[37] Savitz SI, Rivlin MM, Savitz MH (2002). The ethics of prophylactic antibiotics for neurosurgical procedures. J Med Ethics.

[38] Corneli A, Perry B, Collyar D (2018). Assessment of the Perceived Acceptability of an Early Enrollment Strategy Using Advance Consent in Health Care-Associated Pneumonia. JAMA Netw Open.

[39] Greenberg RG, Gamel B, Bloom D (2018). Parents’ perceived obstacles to pediatric clinical trial participation: Findings from the clinical trials transformation initiative. Contemp Clin Trials Commun.

[40] Kyaw L, Pereira NK, Ang CX (2020). Parental preferences in treatment of acute uncomplicated appendicitis comparing surgery to conservative management with antibiotics and their views on research participation. Eur J Pediatr.

[41] Criscione LG, Sugarman J, Sanders L (2003). Informed consent in a clinical trial of a novel treatment for rheumatoid arthritis. Arthritis & Rheum.

[42] Kenyon S, Dixon-Woods M, Jackson CJ (2006). Participating in a trial in a critical situation: a qualitative study in pregnancy. Qual Saf Health Care.

[43] Sherratt FC, Beasant L, Crawley EM (2020). Enhancing communication, informed consent and recruitment in a paediatric urgent care surgical trial: a qualitative study. BMC Pediatr.

[44] Tarrant C, Jackson C, Dixon-Woods M (2015). Consent revisited: the impact of return of results on participants’ views and expectations about trial participation. *Health Expect*.

[45] Webster RK, Rubin GJ (2020). The Effect of Positively Framing Side-Effect Risk in Two Different Formats on Side-Effect Expectations, Informed Consent and Credibility: A Randomised Trial of 16- to 75-Year-Olds in England. Drug Saf.

[46] Sureshkumar P, Caldwell P, Lowe A (2012). Parental consent to participation in a randomised trial in children: associated child, family, and physician factors. Clin Trials.

[47] Parker J, Wright D (2021). Terrible choices in the septic child: a response to the PALOH trial round table authors. *J Med Ethics*.

[48] Saadi A, Mahmood A, Sweeney J (2023). What Is the Benefit of Adding Placebo Side-Effect Information to Positively Framed Patient Leaflets?. Eur J Health Psychol.

[49] Lois A, Kohler JE, Monsell SE (2023). A Video-Based Consent Tool: Development and Effect of Risk–Benefit Framing on Intention to Randomize. J Surg Res.

[50] Hickey HR, Jones AP, Lenney W (2010). Feasibility study to inform the design of a randomised controlled trial to eradicate Pseudomonas aeruginosa infection in individuals with cystic fibrosis. Trials.

[51] Kirschner KL (2003). The challenges of human subject research in the new millenium. Top Stroke Rehabil.

[52] Tromp K, Zwaan CM, van de Vathorst S (2016). Motivations of children and their parents to participate in drug research: a systematic review. Eur J Pediatr.

[53] Loewenstein GF, Weber EU, Hsee CK (2001). Risk as feelings. Psychol Bull.

[54] Zhang B, Shou Y (2022). Immediate emotions and subjective stakes in risky decision-making under uncertainty. Anxiety, Stress, Coping Abbreviated form: A, S, & C.

[55] Blumenthal-Barby JS, Krieger H (2015). Cognitive biases and heuristics in medical decision making: a critical review using a systematic search strategy. Med Decis Making.

[56] Bobadilla-Suarez S, Love BC (2018). Fast or frugal, but not both: Decision heuristics under time pressure. J Exp Psychol Learn Mem Cogn.

[57] Gilovich T, Griffin D, Kahneman D (2002). Heuristics and biases: the psychology of intuitive judgment.

[58] Bart SM, Rubin D, Kim P (2021). Trends in Hospital-Acquired and Ventilator-Associated Bacterial Pneumonia Trials. Clin Infect Dis.

[59] Patterson JK, Pant S, Jones DF (2021). Informed consent rates for neonatal randomized controlled trials in low- and lower middle-income versus high-income countries: A systematic review. PLoS One.

[60] Fehr A, Nieto-Sanchez C, Muela J (2021). From informed consent to adherence: factors influencing involvement in mass drug administration with ivermectin for malaria elimination in The Gambia. Malar J.

[61] Manafa O, Lindegger G, Ijsselmuiden C (2007). Informed consent in an antiretroviral trial in Nigeria. IJME.

[62] Munalula-Nkandu E, Ndebele P, Siziya S (2015). To What did They Consent? Understanding Consent Among Low Literacy Participants in a Microbicide Feasibility Study in Mazabuka, Zambia. Dev World Bioeth.

[63] Carazo Perez S, Folkesson E, Anglaret X (2017). Challenges in preparing and implementing a clinical trial at field level in an Ebola emergency: A case study in Guinea, West Africa. PLoS Negl Trop Dis.

[64] Morin S, Segafredo G, Piccolis M (2023). Expanding access to biotherapeutics in low-income and middle-income countries through public health non-exclusive voluntary intellectual property licensing: considerations, requirements, and opportunities. Lancet Glob Health.

[65] Lahey T (2013). The ethics of clinical research in low- and middle-income countries. Handb Clin Neurol.

[66] NIMHD Diversity and Inclusion in Clinical Trials.

[67] Songstad NT, Roberts CT, Manley BJ (2018). Retrospective Consent in a Neonatal Randomized Controlled Trial. Pediatrics.

