# Supplementary material for: Informed consent and risk communication challenges in antimicrobial clinical trials: a scoping review
Source: BMJ Open. 2024 Nov 24;14(11):e082096. doi: 10.1136/bmjopen-2023-082096 (PMC11590817; doi:10.1136/bmjopen-2023-082096)
Supplement: online supplemental file 1 [file bmjopen-14-11-s001.pdf]

Supplement 1

Search Strategies (26 April 2023)

Published since 2010

Embase (including embase and medline):
(risk* OR uncertain* OR 'risk'/exp OR 'uncertainty'/exp OR 'side effect'/exp OR 'adverse event'/exp OR 'harm*':ab,ti) AND ('information sheet*':ab,ti OR 'information leaflet*':ab,ti OR 'information form*':ab,ti OR consent*:ab,ti OR 'informed':ab,ti OR 'informed consent'/exp) AND trial*:ab,ti AND (antibiotic*:ab,ti OR antibacterial*:ab,ti OR antiviral*:ab,ti OR antiinfective*:ab,ti OR 'anti biotic*':ab,ti OR 'anti bacterial*':ab,ti OR 'anti viral*':ab,ti OR 'anti infective*':ab,ti OR antimicrobi*:ab,ti OR antifung*:ab,ti OR antiparasit*:ab,ti OR 'antiinfective agent'/exp) AND ([article]/lim OR [article in press]/lim OR [review]/lim) AND [english]/lim AND [2010-2023]/py

CINAHL
Limiters - Published Date: 20100101-20231231; Exclude Pre-CINAHL; Exclude MEDLINE records; Language: English; Peer Reviewed
((TI ('information sheet*' OR 'information leaflet*' OR 'information form*' OR consent* OR informed) OR AB ('information sheet*' OR 'information leaflet*' OR 'information form*' OR consent* OR informed) OR (MM "Consent (Research)"))) AND ((TI trial* OR AB trial*)) AND (TX (risk* OR uncertain*) OR TI ('side effect*' OR 'side reaction*' OR 'adverse effect*' OR 'adverse event*' OR 'adverse reaction*' OR harm*) OR AB('side effect*' OR 'side reaction*' OR 'adverse effect*' OR 'adverse event*' OR 'adverse reaction*' OR harm*) OR (MM "Uncertainty") OR (MH "Adverse Drug Event+") OR (MM "Medication Side Effects (Saba CCC)")) AND ((TI (antibiotic* OR antibacterial* OR antiviral* OR antiinfective* OR anti-biotic* OR anti-bacterial* OR anti-viral* OR anti-infective* OR antimicrobi* OR antifung* OR antiparasit*) OR AB (antibiotic* OR antibacterial* OR antiviral* OR antiinfective* OR anti-biotic* OR anti-bacterial* OR anti-viral* OR anti-infective* OR antimicrobi* OR antifung* OR antiparasit*) OR (MH "Antiinfective Agents+")))

PsychInfor (OVID)

1	(antibiotic* or antibacterial* or antiviral* or antiinfective* or anti-biotic* or anti-bacterial* or anti-viral* or anti-infective* or antimicrobi* or antifung* or antiparasit*).mp. [mp=title, abstract, heading word, table of contents, key concepts, original title, tests & measures, mesh word]
2	(harm* or 'adverse effect*' or 'adverse event*' or 'adverse reaction*').mp. [mp=title, abstract, heading word, table of contents, key concepts, original title, tests & measures, mesh word]
3	exp "side effects (drug)"/ or exp "side effects (treatment)"/ or exp Uncertainty/
4	(risk* or uncertain*).af.
5	2 or 3 or 4
6	('information sheet*' or 'information leaflet*' or 'information form*' or consent* or informed).ab,ti.
7	exp Informed Consent/
8	6 or 7
9	trial*.ab,ti.
10	1 and 5 and 8 and 9
11	limit 10 to (peer reviewed journal and english language and yr="2010 -Current")

Web of Science Core (since 2010)
1: TI=('information sheet*' OR 'information leaflet*' OR 'information form*' OR consent* OR

informed) OR AB=('information sheet*' OR 'information leaflet*' OR 'information form*' OR consent* OR informed)
2: TS=(antibiotic* OR antibacterial* OR antiviral* OR antiinfective* OR anti-biotic* OR anti-bacterial* OR anti-viral* OR anti-infective* OR antimicrobi* OR antifung* OR antiparasit*)
3: TI=(trial*) or AB=(trial*)
4: ALL=(risk* OR uncertain*) OR TS=("side effect*" OR "adverse effect*" OR "adverse reaction*"OR "adverse event*" OR harm*)
5: #4 AND #3 AND #2 AND #1 and Review Article or Article (Document Types) and English (Languages)

3

**Published 2000- 2009**	
**Embase (including embase and medline):** (risk* OR uncertain* OR 'risk'/exp OR 'uncertainty'/exp OR 'side effect'/exp OR 'adverse event'/exp OR 'harm*':ab,ti) AND ('information sheet*':ab,ti OR 'information leaflet*':ab,ti OR 'information form*':ab,ti OR consent*:ab,ti OR 'informed':ab,ti OR 'informed consent'/exp) AND trial*:ab,ti AND (antibiotic*:ab,ti OR antibacterial*:ab,ti OR antiviral*:ab,ti OR antiinfective*:ab,ti OR 'anti biotic*':ab,ti OR 'anti bacterial*':ab,ti OR 'anti viral*':ab,ti OR 'anti infective*':ab,ti OR antimicrobi*:ab,ti OR antifung*:ab,ti OR antiparasit*:ab,ti OR 'antiinfective agent'/exp) AND ([article]/lim OR [article in press]/lim OR [review]/lim) AND [english]/lim AND [2000-2009]/py	
**CINAHL** **Limiters** - Published Date: 20000101-20091231; Exclude Pre-CINAHL; Exclude MEDLINE records; Language: English; Peer Reviewed ((TI ('information sheet*' OR 'information leaflet*' OR 'information form*' OR consent* OR informed) OR AB ('information sheet*' OR 'information leaflet*' OR 'information form*' OR consent* OR informed) OR (MM "Consent (Research)"))) AND ((TI trial* OR AB trial*)) AND (TX (risk* OR uncertain*) OR TI ('side effect*' OR 'side reaction*' OR 'adverse effect*' OR 'adverse event*' OR 'adverse reaction*' OR harm*) OR AB('side effect*' OR 'side reaction*' OR 'adverse effect*' OR 'adverse event*' OR 'adverse reaction*' OR harm*) OR (MM "Uncertainty") OR (MH "Adverse Drug Event+") OR (MM "Medication Side Effects (Saba CCC)")) AND ((TI (antibiotic* OR antibacterial* OR antiviral* OR antiinfective* OR anti-biotic* OR anti-bacterial* OR anti-viral* OR anti-infective* OR antimicrobi* OR antifung* OR antiparasit*) OR AB (antibiotic* OR antibacterial* OR antiviral* OR antiinfective* OR anti-biotic* OR anti-bacterial* OR anti-viral* OR anti-infective* OR antimicrobi* OR antifung* OR antiparasit*) OR (MH "Antiinfective Agents+")))	
**PsychInfor (OVID)**	
1	(antibiotic* or antibacterial* or antiviral* or antiinfective* or anti-biotic* or anti-bacterial* or anti-viral* or anti-infective* or antimicrobi* or antifung* or antiparasit*).mp. [mp=title, abstract, heading word, table of contents, key concepts, original title, tests & measures, mesh word]
2	(harm* or 'adverse effect*' or 'adverse event*' or 'adverse reaction*').mp. [mp=title, abstract, heading word, table of contents, key concepts, original title, tests & measures, mesh word]
3	exp "side effects (drug)"/ or exp "side effects (treatment)"/ or exp Uncertainty/
4	(risk* or uncertain*).af.
5	2 or 3 or 4
6	('information sheet*' or 'information leaflet*' or 'information form*' or consent* or informed).ab,ti.
7	exp Informed Consent/

8	6 or 7
9	trial*.ab,ti.
10	1 and 5 and 8 and 9
11	limit 10 to (peer reviewed journal and english language and yr="2000 -2009")

Web of Science Core (2000-2009)

1: TI=('information sheet*' OR 'information leaflet*' OR 'information form*' OR consent* OR informed) OR AB=('information sheet*' OR 'information leaflet*' OR 'information form*' OR consent* OR informed)

2: TS=(antibiotic* OR antibacterial* OR antiviral* OR antiinfective* OR anti-biotic* OR anti-bacterial* OR anti-viral* OR anti-infective* OR antimicrobi* OR antifung* OR antiparasit*)

3: TI=(trial*) or AB=(trial*)

4: ALL=(risk* OR uncertain*) OR TS=("side effect*" OR "adverse effect*" OR "adverse reaction*"OR "adverse event*" OR harm*)

5: #4 AND #3 AND #2 AND #1 and Review Article or Article (Document Types) and English (Languages)
