# Supplementary material for: Informed consent and risk communication challenges in antimicrobial clinical trials: a scoping review
Source: BMJ Open. 2024 Nov 24;14(11):e082096. doi: 10.1136/bmjopen-2023-082096 (PMC11590817; doi:10.1136/bmjopen-2023-082096)
Supplement: Supplementary file 2 [file bmjopen-2023-082096.reviewer_comments.pdf]

PEER REVIEW HISTORY

BMJ Open publishes all reviews undertaken for accepted manuscripts. Reviewers are asked to complete a checklist review form and are provided with free text boxes to elaborate on their assessment. These free text comments are reproduced below.

ARTICLE DETAILS

Title (Provisional)

Informed Consent and Risk Communication Challenges in Antimicrobial Clinical Trials: A Scoping Review

Authors

Shou, Yiyun; Yeo, Joey Elizabeth; Pang, Alexander Shao-Rong; Paterson, David; Mo, Yin

---

VERSION 1 - REVIEW

Reviewer 1

Name Ong, Sean

Affiliation University of Toronto Institute of Health Policy Management and Evaluation

Date 22-Jan-2024

COI None

Shou and colleagues present a broad review looking at issues surround informed consent in clinical trials. The review is well done and covers a broad range of issues, and will be useful to any investigator working within the clinical trials and informed consent space.

Major Comments:

1. I wonder if this is better framed as a scoping review rather than a systematic review. The aims are fairly broad, covering multiple objectives, rather than focusing on a single study question. This is reflected in the inclusion criteria which were designed to be cover a range of topics.

2. In general, the inclusion and exclusion criteria could also benefit from greater focus. For example, in the exclusion criteria, it seems unclear why studies focusing on HIV and tuberculosis were specifically singled out. One inclusion criterion was “in the context of clinical trials involving antibiotic/anti-infective agents”. If the team wanted to focus on studies looking at patients who are acutely unwell or where treatment decisions were urgent, why not state that specifically in inclusion/exclusion criteria?

3. A description of how data were synthesized would be important under the Methods section Was there any quantitative data synthesis or meta-analysis pre-planned? Or was it just narrative synthesis?

4. Table 3 is the main takeaway from this paper. It should thus be elaborated upon much more heavily. Some points are too short or do not give enough information (e.g., “Interest”, “Autonomy”, “Socio-demographic factors”, “Trust in research” – it is not immediately clear to the reader what these points are about, please elaborate).

5. How were the recommendations in Table 4 made? Were these from the reviewed studies, or from the authors’ own input? This needs to be clearer. If it is from the authors own input, could they state how they reached these recommendations? Was it through iterative discussion e.g. a modified Delphi process?

Minor Comments:

1. Page 4, line 8: is “valley of death” quoted from somewhere? What do you mean by this? If quoted, it needs a reference.

2. Page 4, lines 8-12: Reference 1 is an RCT evaluating plazomicin, and is not directly related to the statement made about delay in regulatory approvals and pharmaceutical companies being deterred from investment in antimicrobial drug discovery.

3. Page 4, lines 19-22: It’s not clear how slow recruitment threatens trials’ internal validity and generalizability, please elaborate or remove.

4. The authors provided a link showing their pre-registration of this study https://osf.io/fu49y/ but this is not publicly accessible.

5. Table 3 – the section “Enablers and barriers of consent” is confusing; enablers should probably be put into a separate section from barriers.

**Reviewer**	**2**
**Name**	**Dinh, A**
**Affiliation**	**Versailles Saint Quentin University**
**Date**	**03-May-2024**
**COI**	**None**

This article delves into a complex topic, focusing on the challenge of low consent rates in trials, particularly in the context of infectious diseases, a point that should be reflected in the title. Infectious disease trials present unique challenges, such as the unpredictable nature of participant inclusion and the necessity for rapid enrollment.

A distinction should be made between acceptance/understanding and inclusion rates, as they represent different aspects that warrant discussion. Inclusion often hinges more on

confidence rather than mere understanding or agreement. Understanding is closely tied to the investigator's knowledge level and their ability to effectively communicate complex concepts.

Moreover, disparities in inclusion rates may stem from social, cultural, and economic differences across countries, as well as varying disease types. Access to medication and the economic status of patients, particularly in low- and middle-income countries (LMICs), play significant roles.

Several points merit further exploration, including the impact of financial support for enrolled patients, the influence of patients' educational backgrounds, the availability of investigators, adherence to protocols, and the ability of patients to consent based on their health status.

Additionally, the roles of medical agencies and Institutional Review Boards (IRBs) in the consent process warrant discussion. Lastly, there's room for enhancing trial designs to facilitate higher consent rates.

VERSION 1 - AUTHOR RESPONSE

Reviewer: 1

Major Comments:

1. I wonder if this is better framed as a scoping review rather than a systematic review. The aims are fairly broad, covering multiple objectives, rather than focusing on a single study question. This is reflected in the inclusion criteria which were designed to be cover a range of topics.

> **Response**: Thanks for the suggestion. We have updated the title as suggested, in combination with Reviewer 2's suggestion. In addition, we have revised our manuscript throughout to be more focused and clarify that we focus on issues around risk and uncertainty and risk communication in informed consent in antimicrobial clinical trials.

2. In general, the inclusion and exclusion criteria could also benefit from greater focus. For example, in the exclusion criteria, it seems unclear why studies focusing on HIV and tuberculosis were specifically singled out. One inclusion criterion was "in the context of clinical trials involving antibiotic/anti-infective agents". If the team wanted to focus on studies looking at patients who are acutely unwell or where treatment decisions were urgent, why not state that specifically in inclusion/exclusion criteria?

> **Response**: Thank you for this comment. We focused on antibiotic trials in this review because our planned research was centred on bacterial infections, which present distinct challenges compared to HIV and TB. HIV and TB require unique treatment approaches, regulatory considerations, and informed consent processes for bacterial infections, aside from time sensitivity and disease courses such as acute versus

chronic. The nuances of antibiotic resistance, the rapid progression of bacterial infections, and the specific ethical considerations in bacterial disease trials make this focus necessary for our current and planned studies. Including HIV and TB would have broadened the scope beyond our intended focus on antibiotics and the particular challenges associated with bacterial diseases.

3. A description of how data were synthesized would be important under the Methods section Was there any quantitative data synthesis or meta-analysis pre-planned? Or was it just narrative synthesis?

Response: We apologise for the confusion. We have revised our methods section on Page 7 to clarify that we only provided a narrative synthesis of the articles.

4. Table 3 is the main takeaway from this paper. It should thus be elaborated upon much more heavily. Some points are too short or do not give enough information (e.g., “Interest”, “Autonomy”, “Socio-demographic factors”, “Trust in research” – it is not immediately clear to the reader what these points are about, please elaborate).

Response: Thanks very much for the suggestion. We have added more descriptions to those terms either within the text pages 19-20 in the results section or in Table 3.

5. How were the recommendations in Table 4 made? Were these from the reviewed studies, or from the authors’ own input? This needs to be clearer. If it is from the authors own input, could they state how they reached these recommendations? Was it through iterative discussion e.g. a modified Delphi process?

Response. We apologise for the confusion. In the last paragraph on Page 24, we have now clarified that the recommendations are based on the current review. We also acknowledge that more research and efforts are needed in this field to have more systematic and evidence-based guidance of the recommendations.

Minor Comments:

1. Page 4, line 8: is “valley of death” quoted from somewhere? What do you mean by this? If quoted, it needs a reference.

Response: Thank you for this request. We have added the reference on that line: Chorzelski S, et al. Breaking through the wall: enhancing research and development of antibiotics in science and industry. Global Union for Antibiotic Research & Development Initiative; 2015.

2. Page 4, lines 8-12: Reference 1 is an RCT evaluating plazomicin, and is not directly related to the statement made about delay in regulatory approvals and pharmaceutical companies being deterred from investment in antimicrobial drug discovery.

Response: Thank you for this comment. We have updated the references to Eckburg et al. (2022) on tebipenem, which faced delays for regulatory approvals due to inadequate clinical evidence, and Wagenlehner et al. (2024) on cefipime-tanoborbactam, which has not been approved due to downstream regulatory hurdles.

3. Page 4, lines 19-22: It's not clear how slow recruitment threatens trials' internal validity and generalizability, please elaborate or remove.

Response: Thank you for this comment. We have added further elaboration for that part: "Slow recruitment in clinical trials threatens internal validity by increasing the risk of confounding factors, differential attrition, and operational drift, while it compromises generalizability by potentially altering the target population, reducing temporal relevance, and introducing selection bias."

4. The authors provided a link showing their pre-registration of this but this is not publicly accessible.

Response: We have updated to ensure the page is public.

5. Table 3 – the section "Enablers and barriers of consent" is confusing; enablers should probably be put into a separate section from barriers.

Response: Thanks for the comment. We have reworded the section in Table 3 to "Patients' considerations in consenting" to make the description more appropriate and more aligned with our intext description.

Reviewer: 2
Comments to the Author:
This article delves into a complex topic, focusing on the challenge of low consent rates in trials, particularly in the context of infectious diseases, a point that should be reflected in the title. Infectious disease trials present unique challenges, such as the unpredictable nature of participant inclusion and the necessity for rapid enrollment.

Response: Thanks for the comment. We have updated our title.

A distinction should be made between acceptance/understanding and inclusion rates, as they represent different aspects that warrant discussion. Inclusion often hinges more on confidence rather than mere understanding or agreement. Understanding is closely tied to the investigator's knowledge level and their ability to effectively communicate complex concepts.
Response: We thank the reviewer for the insightful comments. Indeed, the inclusion rate is not the same as consent/comprehension in clinical trials. The inclusion rate is yet another very important issue in conducting clinical trials. Although our article focuses on consent and risk communication, we have added more discussion on Page 23 to highlight the importance of needing attention/and discussion on inclusion rates.
Moreover, disparities in inclusion rates may stem from social, cultural, and economic differences across countries, as well as varying disease types. Access to medication and the economic status of patients, particularly in low- and middle-income countries (LMICs), play significant roles.

Response: The reviewer has a very good point. As we have noted in our article, there is a lack of research in the mainstream scientific database on understanding the issues

in LMICs. We have added on Page 23 (the top paragraph) that disparities can be important to address in our discussion section.

Several points merit further exploration, including the impact of financial support for enrolled patients, the influence of patients' educational backgrounds, the availability of investigators, adherence to protocols, and the ability of patients to consent based on their health status. Additionally, the roles of medical agencies and Institutional Review Boards (IRBs) in the consent process warrant discussion. Lastly, there's room for enhancing trial designs to facilitate higher consent rates.

Response: We agree with the reviewer that those factors are all important issues in recruitment, consent and trial conduct. While our review focused on consent and risk communication aspects, we added further discussion to highlight the importance of the roles of trial investigators and regulators (e.g., IRBs) on Page 23 the last paragraph.

Again, we thank the Reviewers for their insightful feedback.

Sincerely,
The Authors

VERSION 2 - REVIEW

Reviewer 1

Name Ong, Sean

Affiliation University of Toronto Institute of Health Policy Management and Evaluation

Date 10-Sep-2024

COI Principal investigator of a trial comparing simplified layered consent vs full-length consent for a clinical trial (the SIMPLY-SNAP trial)

The authors have addressed all my previous comments adequately. The revised manuscript is much improved in its current version and is suitable for publication.
